# Comprehensive assessment of systemic arteriosclerosis in relation to the ocular resistive index in acute coronary syndrome patients

**DOI:** 10.1038/s41598-021-04196-6

**Published:** 2022-02-11

**Authors:** Yasunari Ebuchi, Taiji Nagaoka, Daisuke Fukamachi, Keisuke Kojima, Naotaka Akutsu, Nobuhiro Murata, Yuki Saito, Daisuke Kitano, Harumasa Yokota, Satoru Yamagami, Yasuo Okumura

**Affiliations:** 1 Division of Cardiology, Nihon University Itabashi Hospital, Tokyo, Japan; 2 Division of Ophthalmology, Nihon University Itabashi Hospital, Ohyaguchi-kamicho, Itabashi-ku, Tokyo, 173-8610 Japan

**Keywords:** Cardiology, Medical research, Ageing, Physiology

## Abstract

This study aimed to investigate the relationship between ocular vascular resistance parameters, evaluated by laser speckle flowgraphy (LSFG), and systemic atherosclerosis, renal parameters and cardiac function in acute coronary syndrome (ACS) patients. We evaluated 53 ACS patients between April 2019 and September 2020. LSFG measured the mean blur rate (MBR) and ocular blowout time (BOT) and resistivity index (RI). 110 consequent patients without a history of coronary artery disease who visited ophthalmology as a control group. Significant positive correlations were observed between ocular RI and systemic parameters in ACS patients, including intima-media thickness (r = 0.34, *P* = 0.015), brachial-ankle pulse-wave velocity (r = 0.41, *P* = 0.002), cystatin C (r = 0.32, *P* = 0.020), and E/e’ (r = 0.34, *P* = 0.013). Ocular RI was significantly higher in the ACS group than in the control group in male in their 40 s (0.37 ± 0.02 vs. 0.29 ± 0.01, *P* < 0.001) and 50 s (0.36 ± 0.02 vs. 0.30 ± 0.01, *P* = 0.01). We found that the ocular RI was associated with systemic atherosclerosis, early renal dysfunction, and diastolic cardiac dysfunction in ACS patients, suggesting that it could be a useful non-invasive comprehensive arteriosclerotic marker.

## Introduction

Atherosclerotic diseases have dramatically increased in Japan with the westernization of diet and rapid aging of the population. The risk factors for atherosclerotic disease systemically affect all vasculature regions, as evidenced by coronary artery disease, cerebrovascular disease, and peripheral artery disease, all linked to one another^1^. Atherosclerosis is generally found in large blood vessels, such as the carotid and lower limb arteries, arteriosclerosis is known to occur in small vessels of the kidneys and retina^2,3^ (so called “arteriolosclerosis”). Nonetheless, the possible association between atherosclerosis and other-region arteriosclerosis and retinal microvessels remains unclear. The laser speckle flowgraphy (LSFG) technique has made it possible to non-invasively detect impairment of the ocular microcirculation by measuring blood flow velocity^4^. Among various parameters measured by LSFG, ocular blowout time (BOT) and resistivity index (RI) were used as indicators of vascular resistance or arteriosclerosis of microvessels^5,6^.

Acute coronary syndrome (ACS) or heart failure with preserved ejection fraction (HFpEF) are associated with old age, hypertension, and type II diabetes mellitus, all strongly related to systemic arteriosclerosis^7^. HFpEF increases the left ventricular and systemic arterial stiffness, and promotes diastolic dysfunction^8^. Diastolic dysfunction was associated with coronary artery calcium score, indicating an association with atherosclerosis^9^. Moreover, it has been reported that transient retinopathy (soft exudate) occurred in patients with acute myocardial infarction (AMI) after percutaneous coronary intervention (PCI), suggesting that ACS might be involved in ocular circulation impairment^10,11^. Despite a high systemic atherosclerotic burden in ACS patients, there are few data that investigate whether systemic arteriosclerosis and diastolic function are linked to the ocular RI and BOT values. This study aimed to investigate the association between systemic atherosclerotic parameters (carotid intima-media thickness [IMT] and brachial-ankle pulse-wave velocity [baPWV]), renal biomarkers, and cardiac function assessed by transthoracic echocardiography, and ocular RI and BOT evaluated by LSFG in ACS patients.

## Results

### Patient characteristics

**Figure 1:**
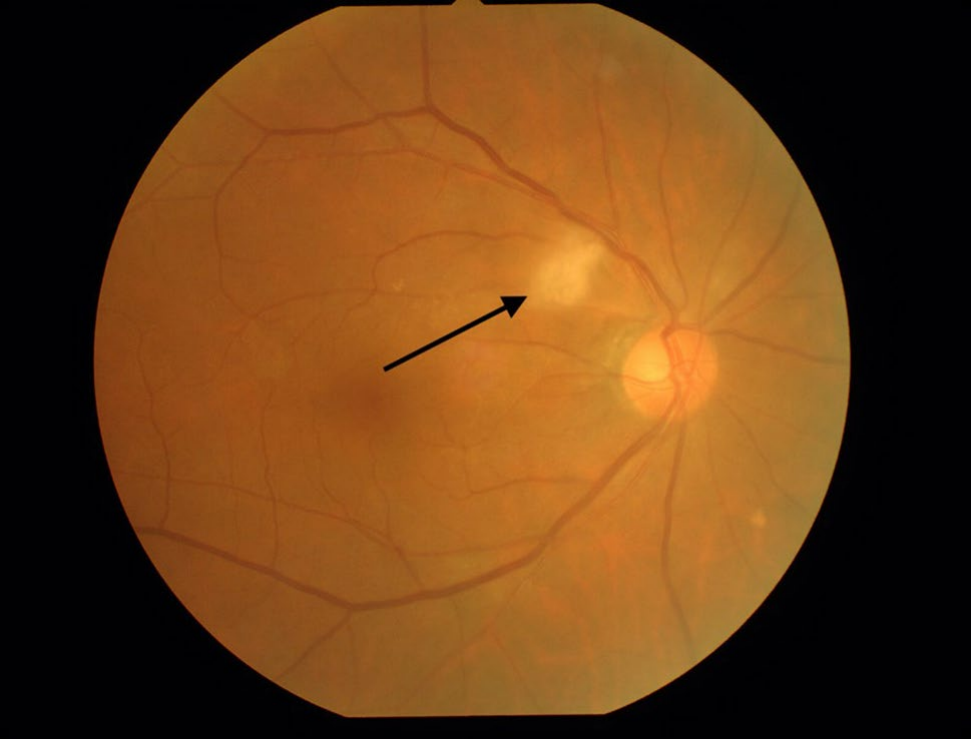
Ophthalmological manifestations 13 days after acute myocardial infarction (AMI). A 77-year-old male was diagnosed with AMI and underwent direct angioplasty 3.5 h after the first episode of severe chest pain. A cotton wool spot (arrow) appeared adjacent to the right optic disc 13 days after AMI. The patient visual acuity was 20/20, and there were no ocular symptoms. The mean ocular blur rate, resistivity index, and blowout time, parameters assessed by laser speckle flowgraphy, were 13.7, 0.58, and 44.1, respectively.

Table 1 summarizes the patient characteristics and the results of optic nerve head (ONH) pulse waveform and transthoracic echocardiographic parameters. A typical fundus image with soft exudate, taken 13 days after AMI, is shown in Fig. 1.

**Table 1:** Characteristic of the patients.

Baseline clinical data	*N* = 53
Age, years	66 ± 13
Male, *n*(%)	47 (89)
Systolic blood pressure, mmHg	114 ± 14
Diastolic blood pressure, mmHg	66 ± 10
Heart rate, beat per minutes	70 ± 8
HTN, *n*(%)	37 (70)
DM, *n*(%)	14 (26)
DLP, *n*(%)	33 (62)
CKD, *n*(%)	8 (15)
Smoking, *n*(%)	38 (72)
STEMI, *n*(%)	39 (74)
SYNTAX score	15 ± 8
Atrial fibrillation, *n*(%)	4 (8)
**Laboratory data**	
BUN, mg/dL	15 (12–20)
Creatinine, mg/dL	0.83 (0.71–1.0)
eGFR, ml/min/1.73 m^2	72.0 ± 22
LDL-Cho, mg/dL	121 ± 35
HDL-Cho, mg/dL	44 (38–50)
TG, mg/dL	113 (68–168)
HbA1c, %	6.0 (5.6–6.7)
NT-ProBNP, pg/mL	285 (64–2518)
Cystatin C, mg/L	0.88 (0.79–1.06)
U-NAG, U/L	7.4 (4.7–15.6)
U-β2MG, µg/L	257 (125–714)
L-FABP, ng/mL	4.7 (1.0–9.8)
U-Creatinine, mg/dL	75 (36–144)
U-Alb, mg/L	40 (23–151)
**Medications**	
ACEI or ARB, *n*(%)	18 (34)
β-blocker, *n*(%)	6 (11)
Statin, *n*(%)	10 (19)
**Systemic atherosclerosis parameter**	
IMT, mm	0.9 (0.75–1.2)
ba-PWV, cm/s	1468 (1232–1881)
**Echocardiographic data**	
LVDd, mm	48 ± 6
LVDs, mm	33 ± 7
LVEF, %	58 ± 10
E, cm/sec	69 (54–87)
A, cm/sec	73 (62–87)
e', cm/sec	6 ± 2
E/e' ratio	11 (9–16)
**Ophthalmic parameters**	
Soft exudate, *n*(%)	21 (40)
Intraocular pressure, mmHg	13 ± 3
Mean MBR	20.0 ± 5.5
Mean BOT	48.9 ± 5.3
Mean RI	0.40 ± 0.10

Values are the mean ± 2SD, median and interquartile range, or *n*(%) of patients.

A, peak mitral A wave velocity; ACEI, angiotensin converting enzyme inhibitor; ARB, angiotensin receptor blocker; baPWV, brachial-ankle pulse-wave velocity; BOT, Blowout time; BUN, blood urea nitrogen; CKD, chronic kidney disease; DM, diabetes mellitus; DLP, dyslipidemia; E peak mitral E wave velocity; e’ peak early diastolic myocardial velocity at septal position recorded by tissue Doppler imaging; E/e’ ratio, ratio of peak mitral E wave velocity to peak early diastolic myocardial velocity at the septal position by tissue Doppler imaging; eGFR, estimated glomerular filtration rate; HbA1c, hemoglobin A1c; HDL-Cho, high-density lipoprotein cholesterol; HTN, hypertension; IMT, intima media thickness; L-FABP, Liver-type Fatty Acid Binding Protein; LVEF, left ventricular ejection fraction; LVDd, left ventricular diastolic dimension; LVDs, left ventricular systolic dimension; LDL-Cho, low-density lipoprotein cholesterol; MBR, mean blur rate; NSTEMI, non ST elevated myocardial infarction; NT-ProBNP, N-terminal pro-brain natriuretic peptide; RI, resistivity index; STEMI, ST elevated myocardial infarction; TG, triglyceride; U-Alb, Urinary albumin excretion; U-β2MG, urinary β2 microgloblin; U-Creatinine, urinary creatinine; U-NAG, urinary N-acetyl-β-D-glucosidase.

*Obtained by Student *t* test, Mann–Whitney *U* test, Chi square test, or Fisher’s exact test, as appropriate.

**Figure 2:**
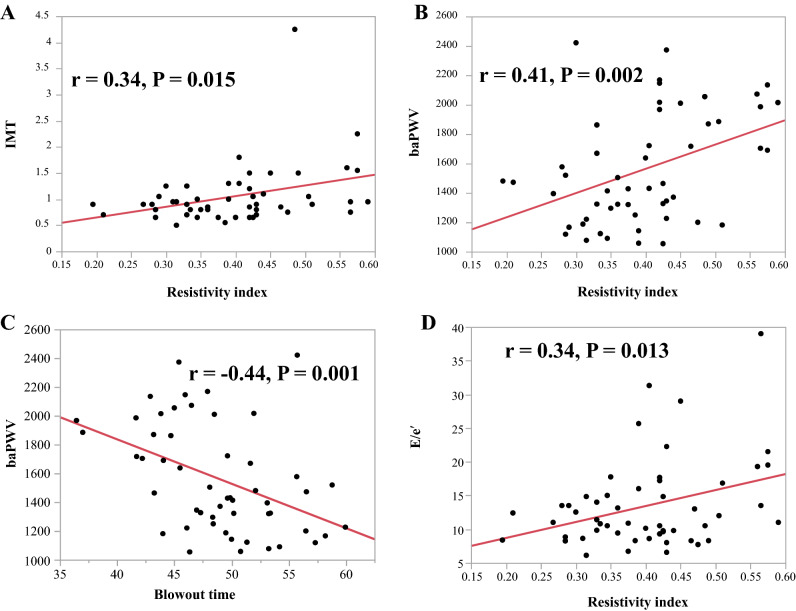
Correlations between resistivity index and intima-media thickness (IMT) (**A**), Brachia-ankle pulse-wave velocity (baPWV) (**B**), and early diastolic velocity to early diastolic myocardial velocity (E/e’) ratio (**D**). Also shown are the correlations between blowout time and baPWV (**C**). The mean RI was positively correlated with the mean IMT (r = 0.34, *P* = 0.015; **A**), baPWV (r = 0.41, *P* = 0.002; **B**) and the E/e’ ratio (r = 0.34, *P* = 0.013; **D**). The mean BOT was negatively correlated with the mean baPWV (r =  −0.44, *P* = 0.001; **C**).

### Relationship between systemic atherosclerosis and mean ocular RI and BOT

**Figure 3:**
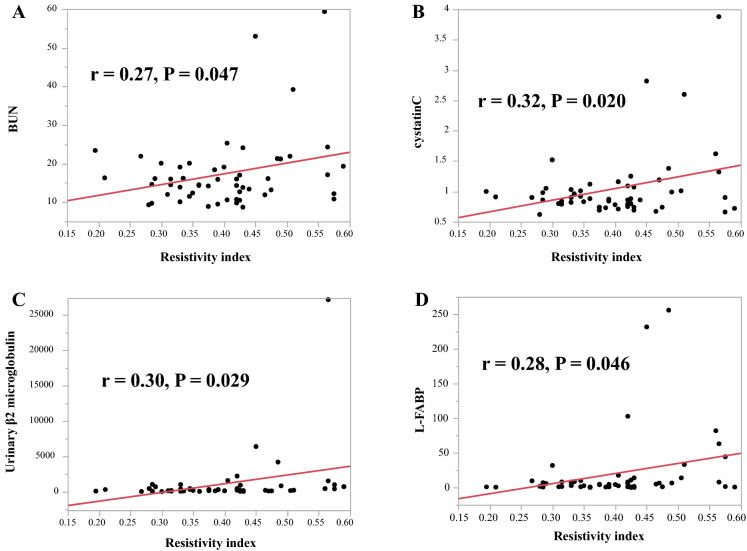
Correlation between the resistivity index and blood urea nitrogen (BUN) (**A**), cystatin C (**B**), Urinary β2 microglobulin (**C**), and liver-type fatty acid-binding protein (L-FABP) (**D**). The mean RI was positively correlated with blood urea nitrogen (BUN; r = 0.27, *P* = 0.047; **A**), cystatin C (r = 0.32, *P* = 0.020; **B**), urinary β2 microglobulin (U-β2MG, r = 0.30, *P* = 0.029; **C**), and liver-type fatty acid-binding protein (L-FABP; r = 0.28, *P* = 0.046; **D**).

Table 2 shows the relationship between systemic atherosclerosis and mean ocular RI and BOT. The mean RI was positively correlated with the mean IMT (r = 0.34, *P* = 0.015; Fig. 3A) and baPWV (r = 0.41, *P* = 0.002; Fig. 3B), but not with laboratory parameters such as low-density lipoprotein cholesterol (LDL-C), triglycerides, and glycosylated hemoglobin (HbA1c) (Table 2). The mean BOT was negatively correlated with the mean baPWV (r =  − 0.44, *P* = 0.001; Fig. 3C). BOT was also not correlated to the laboratory parameters (Table 2).

**Table 2:** Relationship between the ocular RI and BOT and systemic atherosclerosis parameters.

Variables	RI		BOT	
	r	*P* value	r	*P* value
**A. Systemic parameters**				
IMT	**0.34**	**0.015**	−0.25	0.08
baPWV	**0.41**	**0.002**	**−0.44**	**0.001**
LDL-Cho	−0.09	0.53	0.26	0.06
TG	−0.21	0.12	0.14	0.31
HbA1c	0.11	0.45	−0.06	0.69
**B. Renal parameters**				
BUN	**0.27**	**0.047**	−0.22	0.12
Creatinine	0.19	0.16	−0.13	0.36
eGFR	−0.11	0.42	0.18	0.19
Cystatin C	**0.32**	**0.020**	−0.23	0.10
U-NAG	0.03	0.85	0.10	0.49
U-β2MG	**0.30**	**0.029**	−0.21	0.13
U-Creatinine	−0.12	0.39	0.20	0.16
U-Alb	0.17	0.21	−0.25	0.07
L-FABP	**0.28**	**0.046**	−0.22	0.11
**C. Cardiac parameters**				
SYNTAX score	0.19	0.16	**−0.31**	**0.023**
LVDd	0.03	0.82	0.09	0.51
LVDs	−0.01	0.93	0.04	0.75
LVEF	0.17	0.24	−0.06	0.67
LVmass index	0.17	0.24	−0.16	0.25
E/e’	**0.34**	**0.013**	**−0.27**	**0.051**

LVmass index, left ventricular mass index. Other abbreviations are shown in Table 1.

Significant values are in [bold].

### Relationship between renal parameters and mean ocular RI and BOT

**Figure 4:**
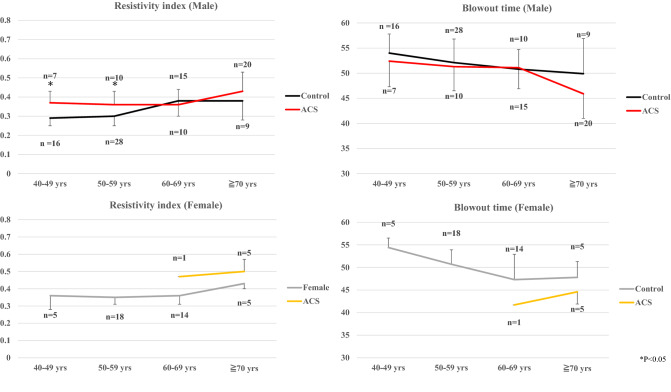
A comparison of the resistivity index (left panels) and blowout time (right panels) between patients with acute coronary syndrome (ACS) and controls in each age category divided by male and female sex. The mean ocular RI was higher in the male ACS patients than in the control male subjects in the 40 to 49 years (0.37 ± 0.02 vs. 0.29 ± 0.01, *P* < 0.001) and 50 to 59 years (0.36 ± 0.02 vs. 0.30 ± 0.01, *P* = 0.01) , while BOT was not different among age categories regardless of sex.

Table 2 shows the relationship between the renal parameters and mean ocular RI and BOT. The mean RI was positively correlated with blood urea nitrogen (BUN; r = 0.27, *P* = 0.047; Fig. 4A), cystatin C (r = 0.32, *P* = 0.020; Fig. 4B), urinary β2 microglobulin (U-β2MG, r = 0.30, *P* = 0.029; Fig. 4C), and liver-type fatty acid-binding protein (L-FABP; r = 0.28, *P* = 0.046; Fig. 4D). No correlation was found with other parameters, including creatine, estimated glomerular filtration rate (eGFR), urinary N-acetyl-β-D-glucosidase (U-NAG), U-creatine, and urinary albumin excretion (U-Alb) (Table 2). The mean BOT was not correlated with any of the renal parameters (Table 2).

### Relationship between cardiac parameters and mean ocular RI and BOT

Table 2 shows the relationship between the mean ocular RI and BOT and cardiac parameters assessed by transthoracic echocardiography. The E/e’ ratio positively correlated with the mean RI (r = 0.34, *P* = 0.013; Fig. 3D) and marginally and negatively with BOT (r =  −0.27, *P* = 0.051; Table 2).

### Differences in systemic atherosclerotic, renal and cardiac parameters, and ocular parameters between ACS patients with and without retinopathy

ACS patients with (*n* = 32) and without (*n* = 21) retinopathy, diagnosed by the existence of soft exudate in the retina, were similar in systemic atherosclerosis, renal, and cardiac parameters. They were also similar in the ocular parameters (RI, 0.40 ± 0.10 vs. 0.41 ± 0.08, *P* = 0.71; BOT, 48.7 ± 5.7 vs. 49.2 ± 4.0, *P* = 0.77).

### Ocular RI and BOT values in ACS patients and controls

**Figure 5:**
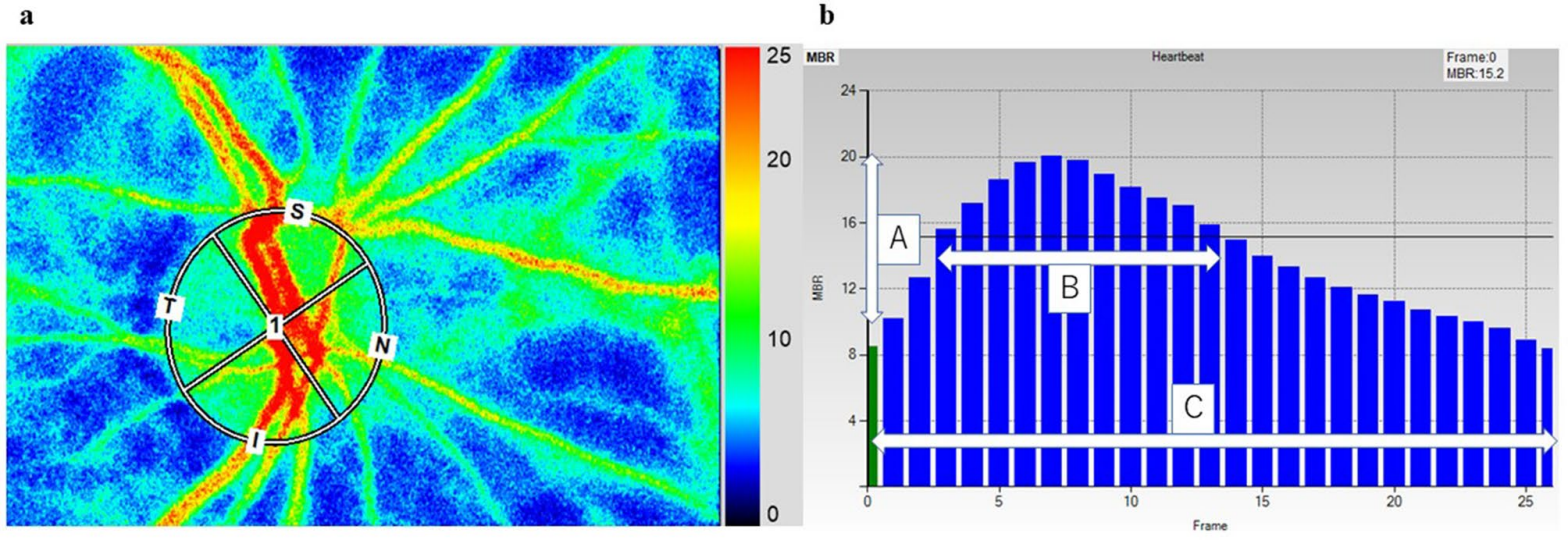
Methods of determining the pulse-wave velocity in the optic nerve head circulation by laser speckle flowgraphy (LSFG). The left panel shows a circle defining the area for measurements in the optic disc area. The right panel shows the normalization of one pulse. A = maximum mean blur rate (MBR)—minimum MBR. B = number of frames spent at one-half the value of A. C = number of frames spent at normalized one pulse.

Table 3 shows the characteristics of the control group subjects (*n* = 110). We compared the RI and BOT values between the ACS and control groups in each age category of 40–49 years, 50–59 years, 60–69 years, and ≥ 70 years, respectively, divided by sex (Fig. 5). Significant differences were found only among males, in the RI values in the 40–49 years and 50–59 years categories.

**Table 3:** Patient characteristics of Control group.

Baseline clinical data	*N* = 110
Age, years	58 ± 9
Male, *n*(%)	68 (62)
HTN, *n*(%)	30 (27)
DM, *n*(%)	5 (5)
DLP, *n*(%)	26 (24)
CKD, *n*(%)	13 (12)
Smoking, *n*(%)	43 (39)

The abbreviations are shown in Table 1.

## Discussion

This study had two main findings. First, the ocular RI obtained by LSFG in ACS patients was significantly associated with atherosclerosis and systemic arteriosclerosis. These trends were similar but weaker for ocular BOT. Second, the RI value in the ACS group was significantly higher in the male aged 40 s to 50 s group than in the same age male control group.

### Clinical value of ocular RI and BOT measured by LSFG

Retinal blood flow is adequately regulated by ocular perfusion pressure and vascular resistance in response to various physiological stimuli^12^. Variations in vascular resistance are generally influenced by vascular endothelial function and smooth muscle elasticity. LSFG allows for quantitative estimation of blood flow in the ONH^13^. The speckle pattern change rates on LSFG are expressed numerically, the flow rate is assessed as MBR, and the RI and BOT are the indexes calculated by MBR waveform analysis^4^. Although these LSFG indexes are the only method that can directly and non-invasively evaluate arteriosclerosis of the microvessels, whether changes in these LSFG indexes are attributable to ocular or systemic (cardiovascular) vascular changes or both remains to be elucidated^3^. This study sheds some light on this question and presents several important findings. The ocular RI correlated positively with systemic (IMT and baPWV), renal (BUN, cystatin C, U-β2MG, and L-FABP), and cardiac (E/e’) parameters, with BOT showing a much weaker association, suggesting that ocular RI may be more appropriate index to systemic parameters than BOT, at least in ACS patients.

Carotid artery assessment by ultrasonography effectively detected the presence of carotid and other atherosclerotic diseases^14^. Increased mean IMT is a major cardiovascular risk factor^15^. baPWV, the most widely used measure of arterial stiffness, was shown to be a strong predictor of future cardiovascular events such as ACS^16^. Previous studies showed that the BOT on LSFG was associated with baPWV and IMT thickening^17^, but the relation between ocular RI and baPWV or IMT was not assessed before. Our results suggest that ocular RI, rather than BOT, could help identify the progression of systemic atherosclerosis in such high burden atherosclerotic patients as ACS patients.

Renal dysfunction is associated with an increased risk of cardiovascular morbidity and mortality. Renal diseases such as chronic kidney disease (CKD) reportedly progress with arteriosclerosis^18,19^. The cardiorenal syndrome is a disorder of the heart and kidney, in which one organ reportedly causes dysfunction of the other. The presence of renal disorder in ACS patients was reported to affect their prognosis^20^. Cystatin C, U-β2MG, and L-FABP are known to be better biomarkers for diagnosing acute kidney disease, which could transition to CKD, than creatinine and eGFR^21^. BOT obtained by LSFG in previous studies was correlated with renal function, and patients with CKD were found to experience an ocular circulatory disorder^22^.

Evaluation of diastolic function after ACS is important because it correlates with infarct size. Diastolic dysfunction has a high risk of death and is associated with poor prognosis independently of left ventricular systolic function^23,24^. The E/e’ ratio is an echocardiographic index used to assess left ventricular diastolic function. It was associated with left ventricular diastolic pressure (and mean pulmonary artery wedge pressure) earlier in the diastole^25^. Previous studies have stated that optic disc BOT obtained by LSFG is significantly correlated with left ventricular diastolic function (E/e’ ratio) in healthy persons^26,27^. Our data showed a similar finding, but the correlation coefficient was higher for RI than for BOT, suggesting that ocular RI assessment after ACS might be useful for determining the presence of diastolic dysfunction.

### Role of transient retinopathy in the ACS patients

We found that 21 (40%) ACS patients had mild and transient retinopathy with soft exudate. Kinoshita et al.^28^ reported that cotton wool spots developed within two months in more than half of the patients with AMI undergoing PCI and then tended to become quiescent without treatment. We found no difference in systemic atherosclerosis, renal, or cardiac parameters between patients with and without soft exudate in our study. Although the patient characteristics in the two studies were different, the results are comparable; the occurrence of soft exudate was unrelated to any ocular parameters measured by LSFG. The soft exudate is known as the representative of ischemic disorders in the local site of the ocular artery. Therefore, our findings and those of this previous study suggest that it may not always directly reflect systemic vascular resistance or atherosclerosis, especially for ACS patients in the subacute phase.

### Clinical implications

Our results indicated a clinical importance that the non-invasive evaluation of ocular blood flow may be a robust marker for promoting the comprehensive evaluations for not only retinal but also systemic arteriosclerosis in ACS patients. Also, the high RI value in middle age patients of 40–60 years can be a potential to predict the vulnerability to cardiovascular disease.

## Study limitations

This study has several limitations. First, this study was conducted in a single institution; the sample size was small because we focused on ACS patients without hemodialysis or assisted circulation apparatus. Particularly, for the comparison of RI and BOT values between the ACS and control groups, the number of subjects was small in each age category. The subject number who had a significant difference in the RI among of the age categories of 40–59 years (ACS *n* = 17 vs. control *n* = 44) might have been statistically acceptable. Second, because most of our ACS patients were male, sex differences could have had some influence on our results, as reported previously^29^. We at least indicated a descriptive result of comparing the male and female RI and BOT values between the ACS and control groups (Fig. 5) to show the sex difference. Third, control group data were collected from the subjects who had visited the Division of Ophthalmology in our hospital with no ocular disorders in the examined eye, so the absence of cardiovascular disease was assessed by the subject clinical record, but not by CAG or coronary CT image. Detailed coronary information was not obtained because control subjects did not have a history of episodes of ACS. Finally, the dates of a comprehensive assessment by ophthalmic examination and other modalities might have affected our results. We excluded ACS patients with severe systemic conditions such as hemodialysis or assisted circulation apparatus to minimize this effect. In addition, all assessments were performed in ACS patients who were considered to have good physical condition within 1 week after a blanking period following ACS onset.

## Conclusion

Ocular vascular assessment by LSFG in ACS patients revealed that ocular RI was strongly associated with systemic parameters, i.e., atherosclerosis and early renal and cardiac diastolic dysfunction whereas these associations were weak for BOT. Therefore, ocular RI might be a useful non-invasive and comprehensive arteriosclerotic marker in ACS patients.

## Methods

We studied 58 patients admitted to the coronary care unit of Nihon University Itabashi Hospital for ACS between April 1 2019, and September 30 2020. All patients underwent PCI on admission day, followed by a visit to the Department of Ophthalmology (mean, 11 ± 5 days after ACS) once their general conditions were stable. The dates for comprehensive assessment by LSFG, ultrasonographic imaging of the carotid artery, brachial-ankle pulse-wave velocity, and transthoracic echocardiography were set within 1 week between the 4 modalities. Patients were excluded from this study if they had glaucoma, uveitis, optic neuropathy, or retinal or choroidal vascular disease, left ventricular (LV) ejection fraction < 30%, or underwent hemodialysis, assisted circulation apparatus or previous intraocular surgery or if they were unable to visit the Department of Ophthalmology on foot. After filtering, 53 patients met the study criteria. LSFG control data were collected from 110 consecutive healthy subjects without any ocular diseases in the examined eye and no history of cardiovascular disease, from whom informed consent was obtained for the evaluation of LSFG. Control subjects were chosen among normal subjects aged 40 years or older who visited the Division of Ophthalmology in our hospital. The institutional review board of Nihon University Itabashi Hospital approved this cross-sectional study, and all participants provided their informed consent for participating in the study. The study was conducted following the tenets of the Declaration of Helsinki.

### Measurement of the carotid intima-media thickness

One to two weeks after admission for ACS, we performed high-resolution ultrasonographic imaging of the carotid artery with an EUB-8500 device (Hitachi, Co. Ltd., Tokyo, Japan), using B-scan mode and a probe frequency of 7.5 MHz. Measurements were performed with the participants in the supine position, and their head slightly turned away from the sonographer. The procedures involved scanning the near and far walls of the carotid artery 1 cm proximally and distally to the carotid bulb in the longitudinal view, and the average of the maximum values on both sides^30^ was used as the mean IMT for data analysis.

### Measurements of brachial-ankle pulse-wave velocity

The baPWV was measured 1–2 weeks after admission for ACS, using a volume-plethysmographic device (baPWV/ABI; Nihon Colin Co., Tokyo, Japan) that simultaneously recorded heart sounds, electrocardiograms, and blood pressure at the left and right brachia and ankles. After the patients rested for a minimum of 5 min and while lying supine, pulse volume waveforms were recorded non-invasively from over the brachial and tibial arteries, and the two waveforms time delay (T) between the feet was measured. The distance (D) covered by the waves was estimated as the distance measured between the two recording sites. baPWV was calculated as follows: baPWV = D (cm)/T(s).

### Laboratory measurements

The following values were measured during the transport to our emergency department: BUN (mg/dL), creatinine (mg/dL), eGFR (mL/min/1.73 m^2^), LDL-C (mg/dL), high-density lipoprotein cholesterol (mg/dL), triglycerides (mg/dL), HbA1c (%), N-terminal pro-brain natriuretic peptide (pg/mL), cystatin C (mg/L), U-NAG (U/L), U-β2MG (µg/L), urinary creatinine (U-creatinine, mg/dL), U-Alb (mg/L), and L-FABP (ng/mL).

### Echocardiographic parameters

Echocardiographic parameters were obtained during the stable phase (mean, 6 ± 4 days after admission for ACS). Echocardiography was performed with the patient in the supine position, using Vivid 7 or Vivid E9 cardiovascular ultrasonographic systems (GE Healthcare, Milwaukee, WI, USA), operated by experienced sonographers who were blinded to patient data. Echocardiographic measurements were performed following the American Society of Echocardiography guideline^31^. Briefly, LV diastolic diameter (LVDd), LV systolic diameter (LVDs), and left atrial diameter were measured on the parasternal long-axis view. LV ejection fraction was measured using the modified Simpson’s method in apical 4- and 2-chamber views. The LV mass was calculated with the formula derived from the data of the American Society of Echocardiography^32^. The LV mass index was calculated as the ratio of the LV mass-to-body surface area. Transmitral flow velocity curves were recorded to measure the peak early (E) and late (A) diastolic velocities. Tissue Doppler imaging at the mitral annulus level was obtained in the septal position to measure the early (e’) and late (A’) diastolic myocardial velocities, as previously described^28^.

### Ocular fundus examinations

All patients underwent a baseline ophthalmic evaluation by a well-trained ophthalmologist (HY) before the ocular blood flow measurement. All patients had good visual acuity (VA > 20/20) and normal intraocular pressure (IOP < 20 mmHg). IOP was monitored by applanation tonometry (Haag Streit, Bern, Switzerland).

### Laser speckle flowgraphy measurements

After the pupils were dilated with a 0.5% tropicamide eye drop, a commercially available LSFG-NAVI system (Softcare Co., Ltd., Fukutsu, Japan) was used to measure ocular circulation at the ONH. The principles of LSFG were previously described in detail^12^. Briefly, the LSFG images were obtained from a 21° section centered on the optic disc. This observation field comprised 750 pixels (width) × 360 pixels (height). The mean blur rate (MBR) was calculated from the moving erythrocytes illuminated by an 830-nm wavelength diode laser beam. The MBRs were expressed in arbitrary units and were considered an indicator of the relative erythrocyte velocity. A total of 118 MBR images were recorded from the ONH area over 4 s. Using accompanying analysis software (LSFG Analyzer, Version 3.3.3.0; Softcare Co., Ltd., Fukutsu, Japan)^22^, a grayscale map of the still images was automatically created by averaging the MBR images (Fig. 2a). MBR can be determined for the entire ONH area (referred to as MA, “mean MBR of the entire area”) or, separately, for vessels (MV, “mean MBR of vascular area”) and tissue (MT, “mean MBR of tissue area”).

The changing MBR pulse wave corresponding to each cardiac cycle was obtained on the analysis screen and displayed after being normalized to one pulse (Fig. 2b). The pulse wave analysis in the ONH circulation was performed on this screen, as was previously described^13^. Briefly, the RI was calculated as the ratio of the difference between the maximum and minimum MBR (labeled A) to the maximum MBR. The number of frames showing one-half the value of A was designated B, and the number of frames showing one cardiac cycle was labeled C. The following formula: BOT = 100 × (B)/(C) was used to analyze the pulse wave in the optic nerve head circulation^33^. We measured MBR, RI, and BOT of the ONH area twice in each eye, and the average values calculated by the LSFG software were used for statistical analysis. All pulse wave analyses in this study were based on the pulse waveform obtained from the MV (corresponding to the large retinal vessels within the ONH area).

### Statistical analyses

Continuous data are expressed as mean ± standard deviation if normally distributed or as median (interquartile range) if otherwise. Comparisons were performed using Student’s *t*-test or Mann–Whitney *U* test. Categorical data are expressed as numbers and percentages and were compared using the chi-squared test or Fisher’s exact test. Linear regression analysis with Spearman rank-order correlation coefficients was used to assess the correlations between the variable, including systemic atherosclerosis, renal, and transthoracic echocardiography cardiac parameters with the LSFG parameters. Statistical significance was defined as a 2-tailed *P*-value < 0.05. Statistical analyses were performed using JMP Version 14.0 (SAS Institute, Cary, NC, USA).

## Data Availability

The authors confirm that the data supporting the findings of this study are available within the article and its supplementary materials.

## Abbreviations

A: Peak mitral A wave velocity
AMI: Acute myocardial infarction
ARB: Angiotensin receptor blocker
baPWV: Brachial-ankle pulse-wave velocity
BOT: Blowout time
BUN: Blood urea nitrogen
CKD: Chronic kidney disease
E: Peak mitral E wave velocity
e’: Peak early diastolic myocardial velocity at septal position recorded by tissue Doppler imaging
E/e’, ratio: Ratio of peak mitral E wave velocity to peak early diastolic myocardial velocity at the septal position by tissue Doppler imaging
eGFR: Estimated glomerular filtration rate
HbA1c: Hemoglobin A1c
HDL-Cho: High-density lipoprotein cholesterol
HFpEF: Heart failure with preserved ejection fraction
IMT: Intima-media thickness
IOP: Intraocular pressure
L-FABP: Liver-type fatty acid-binding protein
LDL-Cho: Low-density lipoprotein cholesterol
LSFG: Laser speckle flowgraphy
LV: Left ventricular
MBR: Mean blur rate
ONH: Optic nerve head
PCI: Percutaneous coronary intervention
RI: Resistivity index
U-Alb: Urinary albumin excretion
U-β2MG: Urinary β2 microgloblin
U-Creatinine: Urinary creatinine
U-NAG: Urinary N-acetyl-β-D-glucosidase
